# Our young cancer patients talk—we learn

**DOI:** 10.18632/oncotarget.21599

**Published:** 2017-10-07

**Authors:** Andrea Ferrari, Maura Massimino

**Affiliations:** Andrea Ferrari: Pediatric Oncology Unit, Fondazione IRCCS Istituto Nazionale Tumori, Milano, Italy

**Keywords:** adolescents, cancer, story, youth project, model

**Figure 1:**
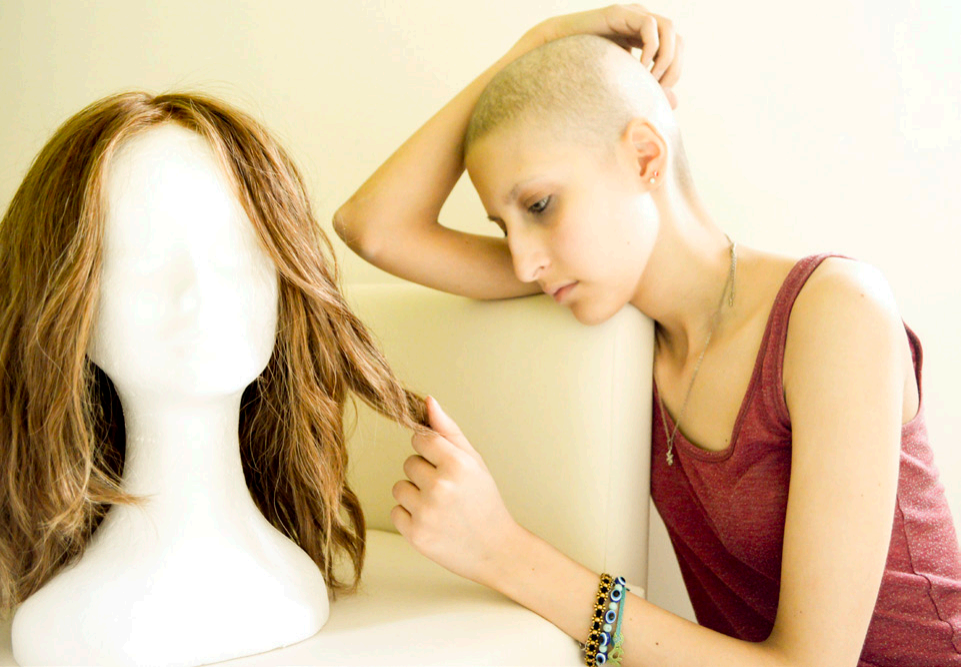
Martina’s self-portrait in “Searching for happiness”.

Adolescents and young adults (AYA) with cancer have attracted more attention in recent years, and the international scientific community has realized they are special patients who need dedicated programs [1]. Publications about the Youth Project run by the dell’Istituto Nazionale dei Tumori in Milan [2] describe activities based on the arts. Patients wrote and recorded a song, “Clouds of Oxygen”, that voiced their fear of dying, but also of being left alone (“Take me with you, away from here”), but they also sang: “The best feeling of all is knowing you have a future and that it’s in your hands” [3]. They expressed their need for beauty in a fashion collection (“We created beauty not just for us, but for others too. We discovered that our creativity can go beyond the limits imposed by our disease”) [4]. They produced a carol about Christmas in hospital—Christmas Balls [5]—that unexpectedly went viral on social media [6]: they said: “The only present we want is a normal, even boring Christmas”; and explained that their caregivers could offer a hypothesis of normality, prepare the ground, but it was up to the young patients to make sense of their experience (“The real normal is the shape we give things”). They took photographs to illustrate their personal search for happiness (in their mum’s Sunday dinner, or music with friends), or the suffering caused by their bodily changes (see Figure 1, Martina’s self-portrait), or reactions like Sefora’s defiant self-portrait published on the paper “In search for happiness” ([7]), taken in front of the mirror, without her wig, challenging her disease, regaining control of her appearance. These publications all discuss AYA with cancer from a novel perspective: the patients themselves take center stage, both in their projects and in our scientific publications. They tell their own story.

The aim of these few lines is to emphasize two lessons learned from working with AYA on the Youth Project. One concerns our model of global, multidisciplinary care that genuinely considers not only the clinical issues, but also the meaning of a patient’s life. The starting point has to be a “protected” space in hospital. Our AYA with cancer voice their courage and awareness of their condition, but also reveal their fragility. Caregivers dealing with such patients often provide age-specific clinical facilities (clinical trials, fertility-preserving schemes), psychosocial support, dedicated spaces, opportunities for socializing and recreation, or means of expression like those described above. But these young people also need “protection”. Adolescents make fragile patients. They need special psychological support because of the impact of their disease and treatment on their still-developing sense of identity and personality. They often remain fragile after completing their treatment too. Projects devised for them must be delicate (making room for lightheartedness, beauty, and hope), but also stable to help them weather the storm of their disease; and they must be professionally organized, and closely connected to the hospital. An example, to better explain: patients embark on such projects as a group; then at some point one of them may suddenly disappear. The others know why; this is their world, a real world where adolescents develop cancer, and may die. Such projects help bring these patients together and make friends (often forming very strong bonds because of the story they share), but they can also provide opportunities for further suffering. For these youngsters, the pain of losing a friend overlaps with the fear of suffering the same fate, or a sense of guilt about being one of the lucky survivors. Their anguish demands a protective network. Physicians and psychologists must be there for them, ready to provide support whenever necessary. There are no rules on how caregivers should handle such situations, but they must put their heart into it, and have the necessary professional expertise.

Another, more personal aspect worth emphasizing is that doctors need to learn how to engage with these patients. The relationship with our young patients should be based on professional trust, but it should make space for understanding, sharing. We have learned from our patients that there comes a time when clinical trials are not enough; there are other things—hidden smiles and laughs, eyes brimming with tears, eyes that make contact, silences, scribbled notes, a vibrating smartphone, the touch of a hand, little lies and tremendous truths—that we cannot leave to others (psychologists, social workers, youth workers, or nurses). There comes a time when we doctors have to bring into play, along with our expertise, all our humanity, our strengths and weaknesses, as adults who have the enormous privilege of standing alongside (and being able to help) young people in the most difficult time of their lives. We urge our colleagues to “feel” that this time has come. “Be enthusiastic, be creative—but especially—be the best you can*”,* the slogan for the adults working on the Youth Project.

Diverse projects have been dedicated to AYA with cancer in various places [8], but the real common denominator for their success must be the human factor, our ability to believe in them, and to change how we behave.
